# Correction: Establishing a Belgian Nutrition Society (BNS): Filling the Void

**DOI:** 10.1186/s13690-022-01018-7

**Published:** 2023-01-16

**Authors:** Patrice Cani, Peter Clarys, Antoine Clinquart, Stefaan De Henauw, Nathalie Delzenne, Peter Deriemaeker, Caroline Douny, Isabelle Guelinckx, Inge Huybrechts, Lieven Huybregts, Patrick Kolsteren, Carl Lachat, Isabelle Laquiere, Yvan Larondelle, Jef Leroy, Guy Manghuin-Rogister, Christophe Matthys, Patrick Mullie, Jean Neve, Marie-Louise Scippo, Isabelle Sioen, Anne-Marie Remaut, John Van Camp, Stefanie Vandevijvere, Margareta Vansant

**Affiliations:** BNS founding members, Brussels, Belgium

**Correction: Arch Public Health 67, 4 (2009)**

**https://doi.org/10.1186/0778-7367-67-1-4**

Following publication of the original article [1], the authors reported an error in the given name and family name designation in the PDF version of the article, the given names and family names need to be interchanged.

The correct author names have been provided in this Correction.

The original article [1] has been corrected.
